# Health related quality of life associated with extreme obesity in adolescents – results from the baseline evaluation of the YES-study

**DOI:** 10.1186/s12955-020-01309-z

**Published:** 2020-03-05

**Authors:** J. Felix, R. Stark, C. Teuner, R. Leidl, B. Lennerz, S. Brandt, J. von Schnurbein, A. Moss, E. Bollow, E. Sergeyev, Y. Mühlig, S. Wiegand, R. W. Holl, T. Reinehr, W. Kiess, A. Scherag, J. Hebebrand, M. Wabitsch, R. Holle

**Affiliations:** 1 Institute of Health Economics and Health Care Management, Helmholtz Zentrum München - German Research Center for Environmental Health, Ingolstaedter Landstrasse 1, Munich, 85764 Neuherberg Germany; 2 Division of Pediatric Endocrinology and Diabetes, Department of Pediatrics and Adolescent Medicine, University Medical Center Ulm, Ulm, Germany; 3 Division of Pediatric Endocrinology, Department of Medicine, Boston Children’s Hospital, Boston, MA USA; 4 Institute for Epidemiology and Medical Biometry, ZIBMT, Ulm University, Ulm, Germany; 5 Hospital for Children and Adolescents, Department of Women and Child Health, University of Leipzig, Leipzig, Germany; 6 Department of Child and Adolescent Psychiatry, Psychosomatics and Psychotherapy, University Hospital Essen, University of Duisburg-Essen, Duisburg, Germany; 7 Ambulatory Obesity Center, Charité University Hospital Berlin, Berlin, Germany; 8 Vestische Children’s Hospital Datteln, University Witten/Herdecke, Witten, Germany; 9 Institute of Medical Statistics, Computer and Data Sciences, Jena University Hospital, Jena, Germany

**Keywords:** Quality of life, Extreme obesity, Obesity grades, Adolescents, EQ-5D-3 L, DISABKIDS, KINDL^R^, BMI

## Abstract

**Background:**

Obesity can significantly reduce health-related quality of life (HRQoL) and may lead to numerous health problems even in youths. This study aimed to investigate whether HRQoL varies among youths with obesity depending on grade of obesity and other factors.

**Methods:**

For the Youths with Extreme obesity Study (YES) (2012–2014), a prospective multicenter cohort study, a baseline sample of 431 obese and extremely obese adolescents and young adults (age 14 to 24 years, BMI ≥30 kg/m^2^) was recruited at four German university medical centers and one job center. Obesity grade groups (OGG) were defined according to BMI (OGG I: 30–34.9 kg/m^2^, OGG II: 35–39.9 kg/m^2^, OGG III (extreme obesity): ≥40 kg/m^2^). HRQoL was measured with the Euroqol-5D-3 L (EQ-5D-3 L), DISABKIDS chronic generic (DCGM-31) and the KINDL^R^ obesity module. Differences between OGGs were assessed with logistic and linear regression models, adjusting for age, sex, and study center in the base model. In a second regression analysis, we included other characteristics to identify possible determinants of HRQoL.

**Results:**

Three hundred fifty-two adolescents (mean age: 16.6 (±2.4), mean BMI: 39.1 (±7.5) kg/ m^2^) with available HRQoL data were analysed. HRQoL of youths in all OGGs was markedly lower than reference values of non-obese adolescents. Adjusting for age and sex, HRQoL of youths in OGG III significantly impaired compared to OGG I. Youths in OGG III were 2.15 times more likely to report problems with mobility in the EQ-5D-3 L than youths in OGG I. A mean difference of 9.7 and 6.6 points between OGG III and I were found for DCGM-31 and KINDL respectively and 5.1 points between OGG II and I for DCGM-31. Including further variables into the regression models, showed that HRQoL measured by DCGM-31 was significantly different between OGGs. Otherwise, female sex and having more than 4 h of daily screen time were also associated with lower HRQoL measured by DCGM-31 and KINDL.

**Conclusion:**

HRQoL of adolescents with obesity is reduced, but HRQoL of adolescents with extreme obesity is particularly affected. Larger and longitudinal studies are necessary to understand the relation of extreme obesity and HRQoL, and the impact of other lifestyle or socioeconomic factors.

**Trial Registration:**

Clinicaltrials.gov NCT01625325; German Clinical Trials Register (DRKS) DRKS00004172.

## Introduction

Obesity in children and adolescents can lead to high morbidity and elevated mortality later in life [1, 2]. This has become a serious public health issue, because the prevalence of overweight and obesity among children and adolescents in Germany and other countries remains at a high level [3, 4]. Excess weight causes various physical disabilities [5] and psychological problems [6] and substantially increases the risk of developing a number of non-communicable diseases, including cardiovascular disease, cancer and diabetes [5]. The most common short-term consequences of pediatric obesity are of a psychological nature [6]. Psychiatric morbidities, such as mood, anxiety and eating disorders, are substantially elevated in treatment seeking extremely obese adolescents [7].

Previous research shows that being obese in childhood or adolescence is associated with reduced HRQoL but most studies included children and/or youths with overweight [8–10]. Indeed, low HRQoL is listed in recent guidelines as a severe comorbidity that might support the indication of bariatric surgery in this group [11] and an increasing number of adolescents with extreme obesity seek bariatric surgery when other treatment options have failed [12, 13]. In general, there are two approaches to measure HRQoL: Generic instruments are used to compare outcomes across different populations, interventions and diseases. In comparison, disease-specific instruments assess disease states and concerns of diagnostic groups and thus might be more sensitive for the detection of small changes that are important to clinicians or patients [14].

The aim of this study was to investigate, whether HRQoL of adolescents with obesity measured with different instruments varies according to WHO obesity grade. Furthermore, other factors that may have an impact on HRQoL in this population were examined.

## Methods

### Sampling and study population

The Youths with Extreme obesity Study (YES) is a prospective multicenter cohort study aiming to improve the medical care and social support structures for adolescents and young adults (henceforth jointly referred to as adolescents) with obesity in Germany [15]. A baseline sample of 431 obese adolescents aged 14 to 24 years with Body-Mass-Index (BMI) ≥ 30 kg/m^2^ was recruited at four university-based German pediatric medical centers (Berlin, Datteln, Leipzig, and Ulm) and one job center (Essen) between June 2012 and November 2014 [16]. Whereas the medical centers recruited subjects based on measured BMI, in Essen, trained personnel recruited unemployed young subjects based on their body silhouette. BMI was confirmed by measurement prior to enrollment. Further diagnostic and therapeutic procedures were then offered on the premises of the medical- and job centers. Participants with insufficient German language skills to independently understand and answer survey questions were excluded from the study. A more detailed description of participants and data collection has been published elsewhere [15, 16]. The study was conducted in accordance to the Helsinki Declaration and the ICH-GCP guideline. Adult subjects provided written informed consent and parental consent was obtained for subjects under the age of 18 years.

### Health related quality of life

HRQoL was measured with three different instruments, the EuroQol five-dimension (EQ-5D-3 L) questionnaire, the DISABKIDS chronic generic module (DCGM-31) and the KINDL^R^ obesity module [17–19].

### EQ-5D-3 l

The EQ-5D-3 L is a generic instrument to measure HRQoL and consists of two parts. In the descriptive part, subjects rate problems in the five dimensions mobility, self-care, usual activities, pain/discomfort and anxiety/depression as none, moderate or severe [17]. In the evaluation part, subjects rate their current health on a visual analogue scale (EQ-VAS) ranging from 0 to 100. In this study the degree of problems reported in the EQ-5D-3 L dimensions was dichotomized to indicate the presence or absence of problems within the respective dimension. Therefore, participants reporting no problems remained with outcome “no problems”, whereas moderate or severe problems were combined to one outcome indicating “problems” in the respective item.

EQ-VAS values were analyzed as a continuous variable. The adult self-report EQ-5D-3 L has been used in children aged 10 to 15 years and older [20] and the combination of EQ-5D-3 L with child-specific quality of life measures has been recommended elsewhere [20]. General population-based studies have been previously published [21, 22] but there are no German reference values for adolescents from 14 to 24 years. Therefore, we compared our results to unpublished data from a German population survey (W&B Survey), conducted by the Wort & Bild Verlag and IFAK research institute in 2011 [21], which also collected EQ-5D-3 L values for 14 to 24 year old participants.

### DISABKIDS chronic generic module (DCGM)

DISABKIDS was developed in a European Commission funded project aiming to develop standardized HRQoL questionnaires for children and adolescents with chronic health conditions [23]. The DISABKIDS chronic generic module does not apply to a specific chronic disease but chronic conditions in general [19] whereby wording may be changed to address disease-specific quality of life. In this study, the DCGM wording was adapted to specifically address adolescents with obesity. The DCGM-31 consists of 37 Likert-scaled items assigned to six dimensions: independence, emotion, social inclusion, social exclusion, limitation, and treatment (if relevant). Each sub-scale consists of six items except the emotion sub-scale, which has seven items. Thus the sub-scales have scores ranging between six and 30, while the subscale “emotion” ranges from seven to 35, if all items of a scale have been completed [19]. Each item indicates the frequency of behaviors or feelings from never (“1”), seldom (“2”), quite often (“3”), very often (“4”) to always (“5”). The scales for negatively worded items were reversed. HRQoL scores were calculated according to the DISABKIDS manual, as the mean of all available questions [19] without considering the medication item (DCGM-31). DISABKIDS scores were then transformed to values between 0 and 100, also as outlined in the handbook of the European DISABKIDS Group [19]. The DCGM-31 has been validated in a Norwegian childhood diabetes population [24] for reliability, construct validity, convergent and discriminant validity in children and adolescents with chronic conditions [25]. Further information regarding structure and domains has been published by Schmidt et al. [19]. Reference values for adolescents 13 to 16 years old were reported with a median of 80 and an interquartile range of 68 to 88 [19].

### KINDL^R^ obesity module

The KINDL^R^ obesity module is a disease-specific subscale of the generic KINDL^R^ and was designed to measure HRQoL of obese children and adolescents. It consists of 12 items with 5 answer dimensions (never – seldom – sometimes – often - all the time) allocated to the dimensions physical well-being, emotional well-being, self-esteem, family, friends, and functional aspects [18]. The scales for negatively worded items were reversed and HRQoL values were transformed to values between 0 and 100 as described in the manual [18]. Adolescent reference values for the KINDL^R^, based on a representative German study sample (*n* = 3737, age = 14–17 years), have been reported by Ravens-Sieberer et al. [26].

### Data processing and variables

Subjects were categorized into three obesity grades: grade I: BMI 30 to 34.9 kg/m^2^, grade II: BMI 35 to 39.9 kg/m^2^ and grade III: BMI ≥ 40 kg/m^2^. To confirm eligibility of interested participants, weight and height were measured by trained medical staff. BMI standard deviation score (SDS) was calculated according to German reference values [27].

Covariates included in regression analyses were age, sex and study center, lifestyle variables (physical activity (PA) and screen time), comorbidities (including hypertension, dyslipidemia and dysglycemia), pre-treatment status regarding obesity (inpatient, outpatient or no pretreatment), and parental migration and education status. PA was assessed according to the question “Do you exercise regularly?” (“yes” or “no”) and screen time was dichotomized based on daily time spent with television, video games, movies, gaming console, computer and internet (more or less than 4 hours per day).

The following comorbid conditions or disorders were used as previously defined by Lennerz et al. [16]: Dysglycemia was defined as fasting blood glucose ≥110 mg/dl (6.11 mmol/L) or 2-h postprandial blood glucose ≥140 mg/dl (7.78 mmol/L) [28]. Dyslipidemia was defined as total cholesterol > 200 mg/dl (5.18 mmol/L) or HDL-cholesterol < 35 mg/dl (0.91 mmol/L) or LDL-cholesterol > 130 mg/dl (3.37 mmol/L), or use of lipid lowering medications. Hypertension was defined as a blood pressure ≥ 95th percentile for age, height and sex, according to the fourth report of the National High Blood Pressure Education Program Working Group on Children and Adolescents, or use of antihypertensive drugs [29]. Pretreatment status was specified according to self-reported previous treatment for obesity (as either outpatient or inpatient) or no pretreatment. Migration status of both parents was assessed and the two answers were transformed into a binary variable (“yes” or “no”) indicating whether at least one parent was born abroad or was a foreign citizen. Parental education status was classified according to the three educational tracks in Germany (low education: ≤9 years; medium education: 10–11 years; high education: ≥12 years).

### Statistical analysis

Descriptive statistics were calculated to describe the total study sample and the different obesity subgroups. Only participants with either complete EQ-5D-3 L or DISABKIDS/KINDL^R^ data were included in the analyses. We addressed possible non-response bias by comparing study participants to participants who were excluded, using Chi-squared and Fisher’s exact test (for variables with *n* < 5) for categorical data and Wilcoxon-Mann-Whitney-test for continuous data.

The association between problems in the 5 EQ-5D-3 L dimensions and BMI was analyzed using logistic regression models adjusted for age, sex, obesity grade and study center (Model A). In a second model (Model B), we additionally included physical activity, screen time, pretreatment status, parents´ educational status, parents’ migration status and comorbidities as potential determinants of HRQoL.

A linear regression model was performed to assess the association between continuous numerical HRQoL variables (EQ-VAS score, DISABKIDS and KINDL^R^ sum scores) and the obesity grades and other covariates as mentioned above. All *p*-values < 0.05 were considered significant without adjusting for multiple testing. All analyses were conducted using SAS 9.4 software (SAS institute Inc. 2011, Cary, NC).

### Sensitivity analysis

In a first sensitivity analysis (SA) we excluded all participants that were recruited in the job center in Essen (*n* = 36). These subjects were excluded, since they were not recruited in a medical center and tended to be older and more often classified in obesity grade III. A second SA examined age-specific BMI percentiles (BMI-SDS) as a continuous variable instead of obesity grades in the logistic and generalized linear regression models using the co-variables as in Model A in the main analysis. A third sensitivity analysis examined the linear association of HRQoL variables with BMI groups in model B but adjusted for participants’ education instead of parental education and imputed for missing variables of all covariables. Participants’ education was classified as follows: lowest educational track: dropped out of school or vocational school; middle educational track: in middle school or high school; highest educational track: grammar school with the aim of higher secondary education. Missing values for covariables (pretreatment, comorbidity, physical activity, migration background, screen time, participant education) were assumed to be missing at random and were imputed using single imputation with the Markov-chain Monte-Carlo method. A fourth sensitivity analysis examined the effect of age on the association between BMI and HRQoL. We compared mean adjusted HRQoL values, using model B, according to BMI group and age below 17 years and above or equal to 17 years.

## Results

### Descriptive statistics

Of 431 participants of the YES study, 352 adolescents (*n* = 180; 51% females) participants with an average age of 16.7 (standard deviation (SD): ±2.4) years had complete data for either EQ-5D or DISABKIDS/KINDL and were thus included in the analyses (Table 1).

**Table 1:** Description of the YES cohort characteristics by obesity grade (means (SD) or frequencies (%))

	Total sample	Obesity grade I (BMI 30–35 kg/m^2^)	Obesity grade II (BMI 35–40 kg/m^2^)	Obesity grade III (BMI > 40 kg/m^2^)
**N (%)**	352 (100.0)	122 (34.7)	100 (28.4)	130 (36.9)
**Age (years)**	16.7 (2.4)	15.8 (1.9)	16.2 (1.9)	17.9 (2.7)
**Gender (female (%))**	180 (51.1)	66 (54.1)	50 (50.0)	64 (49.2)
**BMI (kg/m**^**2**^**)**	39.1 (7.5)	32.6 (1.4)	37.4 (1.4)	46.5 (7.1)
**BMI-SDS**	3.0 (0.5)	2.5 (0.2)	3.0 (0.2)	3.5 (0.4)
**Study centers**				
Berlin	153 (43.5)	68 (55.7)	46 (46.0)	39 (30.0)
Datteln	46 (13.1)	14 (11.5)	19 (19.0)	13 (10.0)
Essen	36 (10.2)	2 (1.6)	2 (2.0)	32 (24.6)
Leipzig	34 (9.7)	9 (7.4)	13 (13.0)	12 (9.2)
Ulm	83 (23.6)	29 (23.8)	20 (20.0)	34 (26.2)
**Cigarette smoking (%)**	54 (16.5)	11 (9.7)	19 (20.4)	24 (19.7)
**Alcohol use (%)**	108 (33.4)	30 (27.5)	27 (29.4)	51 (41.8)
**Obesity Pretreatment status (%)**				
No pretreatment	118 (37.0)	47 (42.7)	32 (36.4)	39 (32.2)
Inpatient	112 (35.1)	30 (27.3)	24 (27.3)	58 (47.9)
Outpatient	89 (27.9)	33 (30.0)	32 (36.4)	24 (19.8)
**Comorbidities (yes (%))**^a^	209 (66.8)	64 (56.6)	59 (62.8)	86 (81.1)
**No regular physical activity (%)**^b^	131 (40.4)	32 (29.4)	37 (39.4)	62 (51.2)
**Screen time > 4 h/d (%)**	199 (61.0)	66 (58.9)	48 (52.2)	85 (69.7)
**Migration background (%)**^c^	159 (47.3)	57 (49.1)	47 (49.5)	55 (44.0)
**Parental education (%)**^d^				
low	98 (31.8)	28 (25.5)	32 (36.0)	38 (34.9)
middle	124 (40.3)	43 (39.1)	37 (41.6)	44 (40.4)
high	86 (27.9)	39 (35.5)	20 (22.5)	27 (24.8)
**Participant education (%)**^e^				
low	84 (25.0)	17 (14.7)	22 (23.2)	45 (36.0)
middle	176 (52.4)	64 (55.2)	53 (55.8)	59 (47.2)
high	76 (22.6)	35 (30.2)	20 (21.1)	21 (16.8)
**No parent full-time employed (%)**	112 (35.3)	40 (37.4)	28 (31.1)	44 (36.7)
**EQ-5D-3 L any problems (%)**				
Mobility (*n* = 349)	63 (18.1)	17 (13.9)	15 (15.2)	31 (24.2)
Self-care (*n* = 348)	6 (1.7)	2 (1.6)	1 (1.0)	3 (2.4)
Usual activities (*n* = 349)	63 (18.1)	19 (15.6)	14 (14.1)	30 (23.4)
Pain/Discomfort (*n* = 349)	159 (45.6)	49 (40.2)	42 (42.4)	68 (53.1)
Anxiety/Depression (*n* = 348)	109 (31.3)	37 (30.3)	28 (28.3)	44 (34.7)
> 1 problem	113 (32.1)	34 (27.9)	27 (27.0)	52 (40.0)
No problem	136 (38.6)	51 (41.8)	43 (43.0)	42 (32.3)
**EQ-VAS** (*n* = 339)	71.8 (22.6)	75.3 (20.7)	71.3 (24.1)	68.9 (23.0)
**DCGM-31** (*n* = 272)	65.1 (17.1)	70.7 (17.8)	65.8 (14.9)	59.3 (16.3)
**DCGM-31 dimensions**				
Independence	67.7 (19.0)	71.5 (19.4)	68.4 (18.5)	63.6 (18.4)
Physical restrictions	69.1 (20.3)	75.0 (20.3)	71.6 (17.6)	61.5 (20.0)
Emotion	51.5 (25.4)	56.7 (27.1)	50.3 (23.6)	47.5 (24.5)
Social exclusion	72.2 (20.9)	76.9 (20.7)	72.7 (20.1)	67.2 (20.7)
Social inclusion	65.4 (17.9)	69.3 (18.8)	67.4 (15.3)	60.2 (17.9)
**KINDL obesity module** (*n* = 316)	60.4 (18.2)	63.6 (18.8)	61.2 (17.7)	56.7 (17.4)

^a^ hypertension, dyslipidemia or dysglycemia

^b^ based on answers to the question “Do you exercise regularly?”;

^c^ at least one parent born abroad and/or foreign citizen status

^d^ low education: ≤9 years; medium education: 10–11 years; high education: ≥12 years

^e^ lowest educational track: dropped out of school or vocational school; middle educational track: middle school; highest educational track: grammar school with the aim of higher secondary education

We observed no evidence for differences between study participants and participants who were excluded because of missing HRQoL data (see supplementary Table 1).

Study participants had a mean BMI of 39.1 (SD: ±7.5) kg/m^2^ and 36.9% were classified as obesity grade III. Participants with obesity grade III had a higher mean age (17.9 years [group III] vs. 16.2 years [group II] vs. 15.8 years [group I]), more frequently reported more than four hours of screen time per day (70% vs. 52% vs. 59% respectively), more frequently reported drinking alcohol (42% vs. 30% vs. 27% respectively) and more frequently reported being physically inactive (51% vs. 39% vs. 29% respectively) than participants with other obesity grades.

In comparison to available reference data from non-obese populations of adolescents, all three instruments showed markedly impaired HRQoL in our obese sample. Reference values for DCGM-31 are reported as a median of 80 and an interquartile range of 68 to 88 [19], whereas in our sample a median of 65.1 (interquartile range 54 to 79) was observed. The mean KINDL^R^ value of our study sample was 60.4 (SD: ±18.2) and thus significantly lower than the reported reference mean of 71.3 [26].

Only 38.6% of the study participants reported no problems on the EQ-5D-3 L but severe problems in the dimensions of the EQ-5D were rarely reported (mobility: *n* = 3; self care: *n* = 1; usual activities: *n* = 2; pain/discomfort: *n* = 8; anxiety/depression: *n* = 15). The mean EQ-VAS score was 71.8 (SD: ±22.6) (Table 1). In comparison, data from 242 adolescents and young adults from the W&B survey (average age and SD: 19.3 ± 3.2 years, 46.3% female, mean BMI and SD: 22.8 ± 3.2) showed that 92.1% reported no problems in any of the EQ-5D-3 L dimensions and mean EQ-5D-3 L EQ-VAS and SD was 93.7 ± 7.8 (supplementary Table 1).

### Results of the logistic regression analysis

Logistic regression (model A) showed higher rates of problems with mobility in participants with obesity grade III compared to obesity grade I (Odds Ratio (OR): 2.15; 95% confidence interval (95%CI): 1.07–4.32). Including further potential determinants (model B) resulted in a non-significant association between obesity grades and reported problems in EQ-5D-3 L dimensions. In this model, daily screen time of more than four hours was associated with more reported problems in the dimensions “usual activities” (OR: 2.29; 95%CI: 1.01–5.21) and “anxiety/depression” (OR: 2.26; 95%CI: 1.16–4.41), and an overall increased likelihood to report at least one problem in the EQ-5D-3 L dimensions (OR: 1.95; 95%CI: 1.08–3.51) (Table 2).

**Table 2:** Logistic regression analysis of the association of obesity grade with problems in the EQ-5D using two models

Variable	Any problems in EQ-5D dimension				
	Mobility OR [95% CI]	Usual Activities OR [95% CI]	Pain/Discomfort OR [95% CI]	Anxiety/Depression OR [95% CI]	at least 1 problem in any dimension OR [95% CI]
**Modell A**^e^					
**Obesity grade**					
I	Ref.	Ref.	Ref.	Ref.	Ref.
II	1.11 [0.52; 2.37]	0.80 [0.37; 1.73]	1.15 [0.66; 2.01]	0.90 [0.49; 1.65]	0.95 [0.54; 1.66]
III	**2.15 [1.07; 4.32]**	1.38 [0.68; 2.78]	1.65 [0.94; 2.89]	1.18 [0.65; 2.12]	1.43 [0.79; 2.58]
**Age**	1.02 [0.87; 1.19]	**1.18 [1.01; 1.38]**	1.07 [0.94; 1.21]	1.09 [0.96; 1.24]	**1.21 [1.05; 1.40]**
**Gender (female)**	0.90 [0.51; 1.57]	0.71 [0.40; 1.24]	**1.70 [1.10; 2.65]**	**2.37 [1.46; 3.84]**	**1.64 [1.04; 2.59]**
**Modell B**^e^					
**Obesity grade**					
I	Ref.	Ref.	Ref.	Ref.	Ref.
II	0.92 [0.36; 2.39]	0.83 [0.33; 2.08]	0.98 [0.50; 1.90]	0.96 [0.45; 2.06]	0.90 [0.45; 1.78]
III	1.90 [0.79; 4.60]	1.46 [0.62; 3.47]	1.87 [0.94; 3.72]	1.28 [0.60; 2.73]	1.63 [0.77; 3.45]
**Age**	1.04 [0.84; 1.28]	1.22 [0.99; 1.50]	1.02 [0.86; 1.21]	**1.20 [1.00; 1.44]**	**1.26 [1.03; 1.55]**
**Gender (female)**	0.66 [0.32; 1.33]	0.66 [0.33; 1.32]	1.54 [0.89; 2.64]	**3.13 [1.68; 5.83]**	1.66 [0.94; 2.94]
**Pretreatment status**					
No pretreatment	Ref.	Ref.	Ref.	Ref.	Ref.
Inpatient	1.13 [0.49; 2.62]	0.87 [0.39–1.95]	1.43 [0.75; 2.73]	1.26 [0.61; 2.59]	1.12 [0.57; 2.21]
Outpatient	0.97 [0.38; 2.49]	0.54 [0.20–1.41]	1.68 [0.84; 3.37]	0.89 [0.40; 1.96]	1.03 [0.50; 2.11]
**Comorbidities (yes)**^a^	0.82 [0.38; 1.77]	0.80 [0.37–1.73]	1.27 [0.71; 2.27]	0.57 [0.30; 1.08]	0.69 [0.38; 1.27]
**Physical activity (yes)**^b^	0.85 [0.41; 1.74]	0.64 [0.32–1.29]	0.90 [0.51; 1.59]	0.72 [0.39; 1.36]	0.66 [0.36; 1.20]
**Parental education**^c^					
low	Ref.	Ref.	Ref.	Ref.	Ref.
medium	0.93 [0.41; 2.10]	1.06 [0.47; 2.40]	0.76 [0.39; 1.48]	0.93 [0.44; 1.99]	0.55 [0.27; 1.12]
high	0.39 [0.15; 1.02]	0.45 [0.17; 1.16]	0.99 [0.49; 2.00]	1.17 [0.53; 2.57]	0.57 [0.27; 1.21]
**Migration background**^d^	**2.33 [1.08; 5.01]**	0.94 [0.46; 1.94]	0.96 [0.54; 1.68]	0.96 [0.51; 1.83]	0.93 [0.51; 1.69]
**Screen time (> 4 h)**	1.20 [0.55; 2.61]	**2.29 [1.01; 5.21]**	1.25 [0.71; 2.21]	**2.26 [1.16; 4.41]**	**1.95 [1.08; 3.51]**

^a^ hypertension, dyslipidemia and dysglycemia

^b^ based on answers to the question “Do you exercise regularly?”;

^c^ low education: ≤9 years; medium education: 10–11 years; high education: ≥12 years

^d^ at least one parent born abroad and/or foreign citizen status

^e^ Both models were additionally adjusted for study centers

Note: Values in bold are significant at *p* < 0.05; Obesity Grade definitions: I: BMI 30 to 34.9 kg/m^2^; II: BMI 35 to 39.9 kg/m^2^; III: BMI ≥ 40 kg/m^2^

### Results of the linear regression analysis

The examination of the association between continuous HRQOL scores and obesity grades in regression model A revealed that subjects with obesity grade III had significantly lower scores in KINDL^R^ and obesity grade was significantly associated with DCGM-31 score. Furthermore, age was negatively associated with EQ-VAS. Adjusting for further potential predictors (model B) resulted in non-significant associations between obesity grades and EQ-VAS and KINDL^R^ scores, however the association between obesity grades and DCGM-31 score remained significant. Screen time of more than four hours a day and female sex were also significantly associated with lower scores of DCGM-31 and KINDL^R^ obesity module (Table 3).

**Table 3:** Linear regression analysis of the association of obesity grade with continuous measures of quality of life using two models

	EQ-VAS	DCGM-31	KINDL^R^
	Estimate [95% CI]	Estimate [95% CI]	Estimate [95% CI]
**Model A**^e^			
**Obesity grade**			
I	Ref.	Ref.	Ref.
II	−3.91 [− 10.05; 2.22]	**− 5.05 [− 9.88; − 0.24]**	− 2.70 [− 7.58; 2.13]
III	− 4.23 [− 10.47; 2.00]	**− 9.70 [− 14.69; − 4.70]**	−6.59 [− 11.63; − 1.55]
**Age**	**−1.45 [− 2.84; − 0.05]**	−0.87 [− 1.97; 0.24]	−0.22 [− 1.43; 0.99]
**Gender (female)**	0.29 [−4.56; 5.14]	**− 8.11 [− 11.91; − 4.31]**	**−9.57 [− 13.47; − 5.67]**
**Model B**^e^			
**Obesity grade**			
I	Ref.	Ref.	Ref.
II	−3.11 [− 10.23; 4.01]	**− 6.48 [− 11.88; − 1.08]**	− 2.41 [− 8.32; 3.51]
III	−4.59 [− 12.00; 2.82]	**−9.65 [− 15.47; − 3.83]**	−5.45 [− 11.66; 0.77]
**Age**	−1.54 [− 3.33; 0.25]	− 0.43 [− 1.88; 1.01]	−0.003 [− 1.67; 1.68]
**Gender (female)**	1.58 [− 4.17; 7.34]	**−9.37 [− 13.76; − 4.98]**	**−9.37 [− 14.17; − 4.56]**
**Pretreatment status**			
No pretreatment	Ref.	Ref.	Ref.
Inpatient	− 0.21 [− 7.11; 6.69]	−3.89 [−9.19; 1.42]	− 2.06 [− 7.75; 3.64]
Outpatient	1.86 [− 5.50; 9.22]	1.34 [−4.45; 7.12]	3.89 [− 2.20; 9.99]
**Comorbidities (yes)**^a^	−1.47 [− 7.70; 4.75]	−1.23 [− 6.00; 3.54]	1.75 [− 3.41; 6.92]
**Physical activity (yes)**^b^	5.64 [− 0.39; 11.68]	5.24 [0.54; 9.95]	4.32 [−0.77; 9.42]
**Parental education**^c^			
low	Ref.	Ref.	Ref.
medium	0.65 [−6.42; 7.72]	1.56 [−4.13; 7.25]	1.78 [−4.21; 7.78]
high	8.52 [1.08; 15.95]	3.25 [−2.64; 9.15]	5.76 [−0.55; 12.08]
**Migration background**^d^	−4.17 [− 10.23; 1.90]	−0.75 [− 5.59; 4.08]	− 2.97 [− 8.14; 2.21]
**Screen time (> 4 h)**	−4.13 [− 10.20; 1.93]	**−5.65 [− 10.38; − 0.92]**	**−5.66 [− 10.75; − 0.57]**

^a^ hypertension, dyslipidemia and dysglycemia

^b^ based on answers to the question “Do you exercise regularly?”;

^c^ low education: ≤9 years; medium education: 10–11 years; high education: ≥12 years

^d^ at least one parent born abroad and/or foreign citizen status

^e^ Both models were additionally adjusted for study centers

Note: Values in bold are significant at *p* < 0.05; Obesity Grade definitions: I: BMI 30 to 34.9 kg/m^2^; II: BMI 35 to 39.9 kg/m^2^; III: BMI ≥ 40 kg/m^2^; DCGM-31: DISABKIDS chronic generic module;

### Sensitivity analysis

In general, results did not change in our SAs. Excluding subjects recruited in the job center in Essen (*n* = 36) from the logistic regression analysis (model A) showed obesity remained negatively associated with problems with mobility (supplementary Table 2). Results of the linear regression analysis without participants from the job center were similar to the results of the main analysis using co-variables of model A. In model B, excluding participants from Essen, obesity grade 3 is significantly associated with KINDL (estimate: -6.70; 95%CI: − 13.09; − 0.31), whereas with Essen, the association is not significant: KINDL (estimate: -5.45; 95%CI: − 11.66; 0.77). The association of BMI groups with DCGM-31 was significant in both the original regression and in the sensitivity analysis (see supplementary Table 3). SA using BMI-SDS instead of obesity grades led to comparable results to the main logistic and linear regression analyses (see supplementary Tables 4 & 5). We also evaluated the effect of adjusting regression analyses with participants’ education instead of parental education. Participants’ education was not associated with EQ-VAS or DCGM-31 but with KINDL^R^ values, but the association of BMI group with the KINDL^R^ values did not change (see supplementary Table 6). A final sensitivity analysis examined the effect of age on the association between HRQoL and BMI groups. Supplemental Figure 1 shows that, while DSGM-31 values are lower for the older participants and BMI groups 2 and 3, the mean values for Kindl^R^ and EQ-VAS are not consistently higher or lower for the older age groups. The overall difference was not significant.

## Discussion

This study analyzed the association between obesity grades and health-related quality of life in adolescents aged 14 to 24 years. In total, we found that HRQoL measured with the DCGM-31 showed significant differences between the three obesity groups and that HRQoL decreased with increasing BMI. EQ-VAS and the KINDL^R^ values also decreased with increasing obesity grades, but the differences were not significant although in all groups HRQoL was clearly impaired compared to available reference values. The impact of extreme obesity was most pronounced in disease-specific HRQoL measures. More than four hours of screen time per day was consistently and significantly associated with a lower HRQoL in all instruments.

Our analyses of EQ-5D-3 L dimensions did not show significant differences between participants in obesity grade I and II with respect to reporting problems. One possible explanation could be a selection bias, such that potential subjects with obesity grade I are more likely to seek treatment and participate in the study if they have a physical or psychological burden of disease that is comparable to grade II subjects. However, compared to healthy-weight groups, HRQoL of obese children and adolescents is found to be significantly impaired [10, 30]. Our results for the DISABKIDS and KINDL^R^ instruments correspond to these findings since they are much lower than reference values [19, 26]. Furthermore, obese adolescents of any obesity grade reported significantly lower EQ-VAS scores compared to adolescents of the W&B survey. Comparing the five dimensions of the EQ-5D-3 L, adolescents with obesity grade III reported problems with mobility in the EQ-5D-3 L more frequently than adolescents with obesity grade I, which confirms previous findings of decreased physical functioning in studies assessing the HRQoL of obese subjects [9, 10, 31].

We also found that gender was associated with problems in the EQ-5D dimensions pain/discomfort and anxiety/depression and with the DCGM-31 and KINDL^R^ obesity module scores. This is consistent with previous research results finding that female adolescents have lower HRQoL values [10, 32]. However, former results also indicated that the effects of BMI are not gender specific [32] and that adolescent girls have lower HRQoL values in general [33].

Our linear regression showed that age was negatively associated with EQ-VAS. This corresponds with previous study results in which obese adolescents reported better HRQoL than obese young adults [34]. Our sensitivity analysis (supplementary Figure 1) showed that KINDL values were least affected by age while inconsistent results for EQ-VAS and DCGM-31 may be in part affected by sample size.

Looking at other variables, we found that adolescents with more than four hours of screen time per day more frequently reported problems with usual activities and anxiety/depression in EQ-5D-3 L and also had lower DCGM-31 and KINDL^R^ scores. Higher screen time may be a compensation for impaired social and emotional functioning as shown for obese adolescents in previous research [30]. Interestingly, PA status was not significantly associated with reported EQ-5D problems or HRQoL scores. Previous research supports a dose-response association between HRQoL and PA level [35]. As obese adolescents are known to be less physically active than normal-weight adolescents [36] and PA status of our study participants was self-reported, it may not have been intense or long enough to positively affect health-related quality of life.

In the extended models, obesity grade was no longer associated with HRQoL measures. However, our cross-sectional analysis does not allow further exploration of potential causal pathways. Higher screen time and less physical activity were clearly associated with obesity grade III but both behaviors can boost obesity as well as result from it.

### Strengths and limitations

To the best of our knowledge, this is one of the few studies focusing directly on HRQoL between different obesity grades of obese adolescents and including more than a hundred participants with extreme (grade III) obesity. Furthermore, the study enables a comparison of generic HRQoL measured with the EQ-VAS, and disease specific HRQoL measured with the DCGM-31 and the KINDL^R^ obesity module.

However, this study also has limitations. First, the sample size of the study population is relatively small. A higher sample size was originally planned but recruitment turned out to be difficult. Thus some of the analyses may lack power, but this is still one of the largest studies available for analysing QOL in youths with extreme obesity. Second, the age range of the study population from adolescents to young adults made the interpretation of results difficult since age of the participants is important for understanding and interpreting HRQOL related questions. In our sensitivity analyses, we found that HRQoL measured by KINDL was not affected by age in our sample, whereas the results of EQ-VAS and DCGM-31 may have been affected by age.

Third, as already reported by Lennerz et al. [16], the majority of study participants were recruited through medical referral and only few participants through specific recruitment efforts or the local job center. Therefore, generalizability may be limited.

Fourth, it could be that using age-specific BMI percentiles (BMI-SDS), as are typically used to identify obesity-related health risks in children and adolescents instead of using absolute BMI, would have led to different results. Since a sensitivity analysis using BMI-SDS instead of BMI-based obesity grades showed similar results in the logistic and in the generalized linear regression analyses, the authors conclude that using absolute BMI to define obesity grades was a practicable method to investigate the association between extreme obesity and HRQoL even in our subgroup of older adolescents. Fifth, the age range of the population compared to the respective questionnaires used could be another limitation. According to the guidelines for DISABKIDS questionnaire users, the DCGM-31 questionnaire was developed for children aged 8–16 years [19], but is also used in young adults in this study. Extreme obesity may affect adolescents, young adults and older adults differently and HRQoL questionnaires may be understood divergently. Our use of the adult self-report version of the EQ-5D-3 L is supported by previous use in another study in children aged 10 to 15 years and older, which found it to be an appropriate instrument for collecting health related quality of life data among injured children [20]. We are aware that the EQ-5D-5 L version is assumed to be more sensitive and it is a validated approach to measure HRQoL in bariatric surgery patients [37]. However, the validation only includes study participants aged 23 or older and would not have been available at the time when planning the present study. The EQ-5D-3 L version has previously been used in representative studies evaluating the impact of obesity on HRQoL [38]. For this reason and given that disease-specific HRQoL measurements are also used in the present study to substantiate EQ-5D results, the authors conclude that the use of the EQ-5D-3 L is justified. Sixth, HRQoL of the extremely obese subjects could only be compared to external data for normal-weight adolescent (not adult) reference groups from a different study population (for EQ-5D) and published reference values (DISABKIDS/KINDL^R^), since a normal weight control group was not included in the study. Furthermore, the wording of the questions of the DISABKIDS questionnaire was changed from generally referring to “illness” to specifically asking about the effect of “overweight” in the individual questions. Although the questionnaire has been previously used in this population [39] the effect of this change in wording was not evaluated with a pilot study or validated.

## Conclusion

The results of this study indicate that the DCGM-31 is the most sensitive instrument to evaluate HRQoL among obese adolescents and young adults. However, compared to normal reference values, all instruments showed that those with extreme obesity suffer a loss in HRQoL and are in highest need of effective weight loss treatment. This impairment increases with age even over the short age range studied here. The increasing prevalence of obesity in adolescents and young adults [40] and the concomitant impairment of HRQoL may contribute to the increasing number of young adults who seek treatment options like bariatric surgery today [12, 13]. Other factors associated with reduced HRQoL in obese adolescents is screen time and being female. To better understand the relation of obesity and HRQoL, more studies including other relevant lifestyle and socioeconomic factors and treatment modalities, as well as longitudinal design studies are needed.

## Supplementary information

**Additional file 1: Table S1.** Description of the YES cohort characteristics compared to non-responders (means (SD) or frequencies (%)) and W&B participants.

**Additional file 2: Table S2.** Logistic regression analysis of the association of obesity grade with problems in the EQ-5D using two models but excluding participants from the job center in Essen.

**Additional file 3: Table S3.** Linear regression analysis of the association of obesity grade with continuous measures of quality of life using two models but excluding participants from the job center in Essen.

**Additional file 4: Table S4.** Logistic regression analysis of the association of BMI-SDS with problems in the EQ-5D, excluding participants from the job center in Essen.

**Additional file 5: Table S5.** Linear regression analysis of the association of BMI-SDS with continuous measures of quality of life using two models but excluding participants from the job center in Essen.

**Additional file 6: Table S6.** Linear regression analysis of the association of obesity grade with continuous measures of quality of life showing the results of model B and using imputed values for missing covariables and adjusting for participant education instead of parental education.

**Additional file 7: Figure S1.** Comparison of mean health related quality of life measured with EQ-VAS, KINDL and DSGM-31 according to BMI group and age below 17 years or above.

## Data Availability

The dataset analysed during the current study is not publicly available because patient consent in this study did not include provision of data for public file sharing.

## Abbreviations

BMI: Body-mass-index
DCGM-31: DISABKIDS chronic generic module without considering the medication item
EQ-5D-3 L: Euroqol- five-dimension three level questionnaire
EQ-VAS: Visual analogue scale
HRQoL: Health-related quality of life
PA: Physical activity
SDS: Standard deviation score
YES: Youths with extreme obesity study
