# Supplementary material for: Health related quality of life associated with extreme obesity in adolescents – results from the baseline evaluation of the YES-study
Source: Health Qual Life Outcomes. 2020 Mar 5;18:58. doi: 10.1186/s12955-020-01309-z (PMC7059717; doi:10.1186/s12955-020-01309-z)
Supplement: Supplementary file 1 — Additional file 1: Table S1. Description of the YES cohort characteristics compared to non-responders (means (SD) or frequencies (%)) and W&B participants. [file 12955_2020_1309_MOESM1_ESM.docx]

| **Supplementary Table 1**: Description of the YES cohort characteristics compared to non-responders (means (SD) or frequencies (%)) and W&B participants | | | |
| --- | --- | --- | --- |
|  | **Responder** | **Non responder** | **W&B survey** |
| **N (%)** | 352 (100) | 79 (100) | 242 (100) |
| **Age (years)** | 16.7 (2.4) | 16.0 (1.7) | 19.3 (3.2) |
| **Gender**, female (%) | 180 (51.1) | 46 (60.5) | 112 (46.3) |
| **BMI (kg/m^2^)** | 39.1 (7.5) | 39.2 (7.6) | 22.8 (3.2) |
| **BMI-SDS** | 3.0 (0.5) | 3.1 (0.5) |  |
| **Study centers** |  |  |  |
| Berlin | 153 (43.5) | 28 (36.8) |  |
| Datteln | 46 (13.1) | 3 (4.0) |  |
| Essen | 36 (10.2) | 17 (22.4) |  |
| Leipzig | 34 (9.7) | 9 (11.8) |  |
| Ulm | 83 (23.6) | 19 (25.0) |  |
| **Comorbidities (yes (%))^1^** | 209 (66.8) |  |  |
| **Monitor time > 4 hours/day (%)** | 199 (61.0) | 33 (57.9) |  |
| **Cigarette smoking (%)** | 54 (16.5) | 11 (19.0) |  |
| **Alcohol use (%)** | 108 (33.4) | 20 (36.4) |  |
| **No regular physical activity (%)^2^** | 131 (40.4) | 21 (36.9) |  |
| **Migration background (%)^3^** | 159 (47.3) | 32 (55.2) |  |
| **Parental education status (%)^4^** |  |  |  |
| low | 98 (31.8) | 18 (36.0) |  |
| middle | 124 (35.2) | 21 (42.0) |  |
| high | 86 (24.4) | 11 (22.0) |  |
| **No parent full-time employed (%)** | 112 (35.3) | 20 (35.1) |  |
| **EQ-5D-3L any problems (%)** |  |  |  |
| Mobility (n=349) | 63 (18.1) |  | 2 (0.8) |
| Self-care (n=348) | 6 (1.7) |  | 1 (0.4) |
| Usual activities (n=349) | 63 (18.1) |  | 1 (0.4) |
| Pain/Discomfort (n=349) | 159 (45.6) |  | 10 (4.1) |
| Anxiety/Depression (n=348) | 109 (31.3) |  | 13 (5.4) |
| > 1 problem | 113 (32.1) |  | 8 (3.3) |
| No problem | 136 (38.6) |  | 223 (92.1) |
| **VAS (EQ-5D-3L)** | 71.8 (22.6) |  | 93.7 (7.8) |

^1^ hypertension, dyslipidemia and dysglycemia

^2^ based on answers to the question “Do you exercise regularly?”;

^3^ at least one parent born abroad and/or foreign citizen status

^4­^ low education: no school graduation, high school with apprenticeship; medium education: middle school apprenticeship; high education: grammar school with/without university attendance

Note: W&B survey: survey of “Wort und Bild” readers in Germany using Euroqol 5 Dimension 3 levels (EQ-5D-3L); VAS: visual analogue scale
