# Supplementary material for: Health related quality of life associated with extreme obesity in adolescents – results from the baseline evaluation of the YES-study
Source: Health Qual Life Outcomes. 2020 Mar 5;18:58. doi: 10.1186/s12955-020-01309-z (PMC7059717; doi:10.1186/s12955-020-01309-z)
Supplement: Supplementary file 2 — Additional file 2: Table S2. Logistic regression analysis of the association of obesity grade with problems in the EQ-5D using two models but excluding participants from the job center in Essen. [file 12955_2020_1309_MOESM2_ESM.docx]

| **Supplementary Table 2** Logistic regression analysis of the association of obesity grade with problems in the EQ-5D using two models but excluding participants from the job center in Essen | | | | | | | | | | |
| --- | --- | --- | --- | --- | --- | --- | --- | --- | --- | --- |
|  |  | **Any problems in EQ-5D dimension** | | | | | | |  |  |
|  | **Variable** | **Mobility**  OR [95% CI] |  | **Usual Activities**  OR [95% CI] |  | **Pain/Discomfort**  OR [95% CI] |  | **Anxiety/Depression**  OR [95% CI] |  | **at least 1 problem**  OR [95% CI] |
| **Modell A^a^** | **Obesity grade** |  |  |  |  |  |  |  |  |  |
|  | I | Ref. |  | Ref. |  | Ref. |  | Ref. |  | Ref. |
|  | II | 1.13 [0.53; 2.42] |  | 0.86 [0.40; 1.87] |  | 1.16 [0.66; 2.03] |  | 0.87 [0.47; 1.63] |  | 0.91 [0.52; 1.60] |
|  | III | 2.18 [1.06; 4.47] |  | 1.46 [0.70; 3.03] |  | 1.65 [0.93; 2.95] |  | 1.17 [0.64; 2.17] |  | 1.42 [0.77; 2.61] |
|  | **Age** | 0.98 [0.82; 1.17] |  | 1.17 [0.98; 1.39] |  | 1.04 0.91; 1.20] |  | 1.16 [1.00; 1.34] |  | 1.20 [1.02; 1.40] |
|  | **Gender (female)** | 0.82 [0.46; 1.47] |  | 0.60 [0.33; 1.10] |  | 1.52 [0.95; 2.41] |  | 2.71 [1.62; 4.55] |  | 1.67 [1.04; 2.68] |
| **Modell B^a^** | **Obesity grade** |  |  |  |  |  |  |  |  |  |
|  | I | Ref. |  | Ref. |  | Ref. |  | Ref. |  | Ref. |
|  | II | 0.91 [0.35; 2.38] |  | 0.92 [0.36; 2.35] |  | 1.00 [0.51; 1.94] |  | 1.03 [0.48; 2.21] |  | 0.92 [0.46; 1.82] |
|  | III | 1.77 [0.71; 4.40] |  | 1.68 [0.68; 4.10] |  | 2.01 [0.94; 3.72] |  | 1.39 [0.65; 3.01] |  | 1.73 [0.81; 3.73] |
|  | **Age** | 1.05 [0.84; 1.32] |  | 1.21 [0.98; 1.51] |  | 1.02 [1.00; 4.05] |  | 1.23 [1.02; 1.49] |  | 1.25 [1.01; 1.54] |
|  | **Gender (female)** | 0.58 [0.28; 1.22] |  | 0.61 [0.30; 1.24] |  | 1.00 [0.84; 1.20] |  | 2.98 [1.59; 5.60] |  | 1.66 [0.93; 2.95] |
|  | **Pretreatment of obesity** |  |  |  |  |  |  |  |  |  |
|  | No pretreatment | Ref. |  | Ref. |  | Ref. |  | Ref. |  | Ref. |
|  | Inpatient | 0.92 [0.38; 2.21] |  | 0.84 [0.36; 1.92] |  | 1.35 [0.70; 2.62] |  | 1.16 [0.56; 2.41] |  | 1.04 [0.52; 2.08] |
|  | Outpatient | 0.88 [0.34; 2.30] |  | 0.54 [0.20; 1.45] |  | 1.61 [0.80; 3.25] |  | 0.87 [0.39; 1.93] |  | 0.96 [0.46; 1.99] |
|  | **Comorbidities (yes)^1^** | 0.81 [0.34; 1.76] |  | 0.78 [0.36; 1.70] |  | 1.27 [0.71; 2.27] |  | 0.56 [0.30; 1.06] |  | 0.69 [0.38; 1.26] |
|  | **Physical activity (yes)^2^** | 0.80 [0.38; 1.69] |  | 0.58 [0.28; 1.19] |  | 0.99 [0.55; 1.75] |  | 0.67 [0.35; 1.26] |  | 0.72 [0.39; 1.34] |
|  | **Parental education ^3^** |  |  |  |  |  |  |  |  |  |
|  | low | Ref. |  | Ref. |  | Ref. |  | Ref. |  | Ref. |
|  | medium | 0.91 [0.39; 2.13] |  | 1.07 [0.45; 2.51] |  | 0.77 [0.39; 1.52] |  | 0.87 [0.40; 1.90] |  | 0.55 [0.26; 1.15] |
|  | high | 0.34 [0.12; 0.95] |  | 0.48 [0.18; 1.30] |  | 0.96 [0.47; 1.96] |  | 1.18 [0.53; 2.63] |  | 0.53 [0.25; 1.16] |
|  | **Migration background^4^** | 2.32 [1.04; 5.18] |  | 0.87 [0.41; 1.85] |  | 0.98 [0.55; 1.75] |  | 0.89 [0.46; 1.71] |  | 0.96 [0.52; 1.77] |
|  | **Screen time (>4h)** | 1.37 [0.62; 3.05] |  | 2.24 [1.00; 5.11] |  | 1.27 [0.72; 2.26] |  | 2.23 [1.14; 4.36] |  | 2.00 [1.10; 3.62] |

^1^ hypertension, dyslipidemia and dysglycemia

^2^ based on answers to the question “Do you exercise regularly?”;

^3^ low education: no school graduation, high school with apprenticeship; medium education: middle school apprenticeship; high education: grammar school with/without university attendance;

^4^ at least one parent born abroad and/or foreign citizen status

^a^ Both models were additionally adjusted for institutes.

Note: Obesity Grade definitions: I: BMI 30 to 34.9 kg/m^2^; II: BMI 35 to 39.9 kg/m^2^; III: BMI ≥ 40 kg/m^2^; EQ-5D: Euroqol 5-dimension questionnaire
