# Supplementary material for: Health related quality of life associated with extreme obesity in adolescents – results from the baseline evaluation of the YES-study
Source: Health Qual Life Outcomes. 2020 Mar 5;18:58. doi: 10.1186/s12955-020-01309-z (PMC7059717; doi:10.1186/s12955-020-01309-z)
Supplement: Supplementary file 3 — Additional file 3: Table S3. Linear regression analysis of the association of obesity grade with continuous measures of quality of life using two models but excluding participants from the job center in Essen. [file 12955_2020_1309_MOESM3_ESM.docx]

**Supplementary Table 3** Linear regression analysis of the association of obesity grade with continuous measures of quality of life using two models but excluding participants from the job center in Essen

|  | Variable | **EQ-VAS** | **DCGM-31** | **KINDLᴿ obesity** |
| --- | --- | --- | --- | --- |
|  |  | Estimate [95% CI] | Estimate [95% CI] | Estimate [95% CI] |
| **Model A** | **Obesity grade** |  |  |  |
|  | I | Ref. | Ref. | Ref. |
|  | II | -3.95 [-10.07; 2.18] | **-5.11 [-10.04; -0.18]** | -2.94 [-7.88; 2.00] |
|  | III | -4.81 [-11.25; 1.64] | **-9.34 [-14.52; -4.17]** | -7.11 [-12.30; -1.93] |
|  | **Age** | **-1.78 [-3.33; -0.23]** | -1.08 [-2.36; 0.20] | -0.28 [-1.66; 1.10] |
|  | **Gender (female)** | 1.43 [-3.68; 6.54] | **-8.24 [-12.31; -4.17]** | **-8.94 [-13.05; -4.83]** |
| **Model B** | **Obesity grade** |  |  |  |
|  | I | Ref. | Ref. | Ref. |
|  | II | -3.87 [-10.94; 3.20] | **-6.69 [-12.15; -1.23]** | -2.47 [-8.41; 3.47] |
|  | III | -6.19 [-13.66; 1.29] | **-9.46 [-15.40; -3.52]** | -6.70 [-13.09; -0.31] |
|  | **Age** | -1.81 [-3.66; 0.04] | -0.66 [-2.21; 0.89] | -0.03 [-1.83; 1.76] |
|  | **Gender (female)** | 2.04 [-3.76; 7.84] | **-9.54 [-14.05; -5.04]** | **-8.73 [-13.61; -3.85]** |
|  | **Pretreatment of obesity** |  |  |  |
|  | No pretreatment | Ref. | Ref. | Ref. |
|  | Inpatient | 1.47 [-5.53; 8.48] | -2.66 [-8.15; 2.84] | -0.87 [-6.74; 4.99] |
|  | Outpatient | 2.22 [-5.16; 9.60] | 1.86 [-3.99; 7.72] | 4.01 [-2.18; 10.20] |
|  | **Comorbidities (yes)^1^** | -1.41 [-7.56; 4.74] | -1.28 [-6.08; 3.53] | 1.89 [-3.27; 7.04] |
|  | **Physical activity (yes)^2^** | 5.84 [-0.27; 11.96] | **5.16 [0.30; 10.02]** | 4.63 [-0.58; 9.84] |
|  | **Parental education^3^** |  |  |  |
|  | low | Ref. | Ref. | Ref. |
|  | medium | 2.38 [-4.85; 9.62] | 2.00 [-3.86; 7.86] | 2.10 [-4.10; 8.30] |
|  | high | 9.07 [1.50; 16.65] | 3.46 [-2.67; 9.59] | 5.17 [-1.36; 11.71] |
|  | **Migration background^4^** | -3.05 [-9.21; 3.11] | -0.56 [-5.48; 4.36] | -2.31 [-7.59; 2.98] |
|  | **Screen time (>4h)** | -4.18 [-10.22; 1.86] | **-5.89 [-10.70; -1.07]** | **-5.52 [-10.65; -0.39]** |

^1^ hypertension, dyslipidemia and dysglycemia

^2^ based on answers to the question “Do you exercise regularly?”;

^3^ low education: no school graduation, high school with apprenticeship; medium education: middle school apprenticeship; high education: grammar school with/without university attendance;

^4^ at least one parent born abroad and/or foreign citizen status

^a^ Both models were additionally adjusted for institutes.

Note: EQ-VAS: visual analogue scale; DCGM-31: DISABKIDS chronic generic module without considering the medication item; Obesity Grade definitions: I: BMI 30 to 34.9 kg/m^2^; II: BMI 35 to 39.9 kg/m^2^; III: BMI ≥ 40 kg/m^2^
