# Supplementary material for: Health related quality of life associated with extreme obesity in adolescents – results from the baseline evaluation of the YES-study
Source: Health Qual Life Outcomes. 2020 Mar 5;18:58. doi: 10.1186/s12955-020-01309-z (PMC7059717; doi:10.1186/s12955-020-01309-z)
Supplement: Supplementary file 4 — Additional file 4: Table S4. Logistic regression analysis of the association of BMI-SDS with problems in the EQ-5D, excluding participants from the job center in Essen. [file 12955_2020_1309_MOESM4_ESM.docx]

| **Supplementary Table 4** Logistic regression analysis of the association of BMI-SDS with problems in the EQ-5D, excluding participants from the job center in Essen | | | | | | | | | | |
| --- | --- | --- | --- | --- | --- | --- | --- | --- | --- | --- |
|  |  | **Any problems in EQ-5D dimension** | | | | | | |  |  |
|  | **Variable** | **Mobility** |  | **Usual Activities** |  | **Pain/Discomfort** |  | **Anxiety/Depression** |  | **at least 1 problem** |
|  |  | OR [95% CI] |  | OR [95% CI] |  | OR [95% CI] |  | OR [95% CI] |  | OR [95% CI] |
| **Modell A^a^** | **BMI-SDS** | 2.39 [1.36; 4.21] |  | 1.75 [1.00; 3.06] |  | 1.42 [0.90; 2.21] |  | 1.26 [0.79; 2.01] |  | 1.54 [0.94; 2.51] |
|  | **Age** | 1.04 [0.89; 1.22] |  | 1.19 [1.02; 1.39] |  | 1.09 [0.96; 1.24] |  | 1.10 [0.96; 1.25] |  | 1.23 [1.07; 1.42] |
|  | **Gender (female)** | 0.91 [0.52; 1.59] |  | 0.74 [0.42; 1.29] |  | 1.69 [1.09; 2.63] |  | 2.39 [1.48; 3.87] |  | 1.64 [1.04; 2.58] |
| **Modell B^a^** | **BMI SDS** | 2.07 [0.98; 4.34] |  | 1.60 [0.78; 3.28] |  | 1.54 [0.88; 2.72] |  | 1.08 [0.59; 2.00] |  | 1.51 [0.80; 2.83] |
|  | **Age** | 1.03 [0.82; 1.29] |  | 1.22 [0.99; 1.50] |  | 1.04 [0.88; 1.23] |  | 1.21 [1.01; 1.45] |  | 1.28 [1.05; 1.57] |
|  | **Gender (female)** | 0.66 [0.32; 1.33] |  | 0.66 [0.33; 1.32] |  | 1.52 [0.89; 2.60] |  | 3.10 [1.67; 5.77] |  | 1.64 [0.93; 2.90] |
|  | **Pretreatment of obesity** |  |  |  |  |  |  |  |  |  |
|  | No pretreatment | Ref. |  | Ref. |  | Ref. |  | Ref. |  | Ref. |
|  | Inpatient | 1.09 [0.47; 2.54] |  | 0.85 [0.38; 1.91] |  | 1.45 [0.76; 2.76] |  | 1.29 [0.63; 2.64] |  | 1.13 [0.57; 2.22] |
|  | Outpatient | 0.92 [0.36; 2.36] |  | 0.52 [0.20; 1.36] |  | 1.63 [0.82; 3.25] |  | 0.88 [0.40; 1.95] |  | 1.00 [0.49; 2.05] |
|  | **Comorbidities (yes)^1^** | 0.82 [0.38; 1.76] |  | 0.81 [0.37; 1.74] |  | 1.30 [0.73; 2.32] |  | 0.58 [0.31; 1.10] |  | 0.70 [0.38; 1.29] |
|  | **Physical activity (yes)^2^** | 0.92 [0.44; 1.92] |  | 0.68 [0.34; 1.38] |  | 0.93 [0.53; 1.65] |  | 0.72 [0.38; 1.36] |  | 0.68 [0.37; 1.25] |
|  | **Parental education ^3^** |  |  |  |  |  |  |  |  |  |
|  | low | Ref. |  | Ref. |  | Ref. |  | Ref. |  | Ref. |
|  | medium | 0.89 [0.40; 2.02] |  | 1.06 [0.47; 2.38] |  | 0.75 [0.38; 1.45] |  | 0.94 [0.44; 2.01] |  | 0.54 [0.26; 1.10] |
|  | high | 0.41 [0.15; 1.07] |  | 0.47 [0.18; 1.21] |  | 1.01 [0.51; 2.03] |  | 1.19 [0.54; 2.59] |  | 0.59 [0.28; 1.25] |
|  | **Migration background^4^** | 2.27 [1.05; 4.90] |  | 0.93 [0.45; 1.92] |  | 0.96 [0.55; 1.69] |  | 0.98 [0.52; 1.87] |  | 0.93 [0.51; 1.69] |
|  | **Screen time (>4h)** | 1.23 [0.57; 2.66] |  | 2.36 [1.04; 5.34] |  | 1.28 [0.73; 2.25] |  | 2.30 [1.19; 4.46] |  | 2.02 [1.13; 3.61] |

^1^ hypertension, dyslipidemia and dysglycemia

^2^ based on answers to the question “Do you exercise regularly?”;

^3^ low education: no school graduation, high school with apprenticeship; medium education: middle school apprenticeship; high education: grammar school with/without university attendance;

^4^ at least one parent born abroad and/or foreign citizen status

^a^ Both models were additionally adjusted for institutes.

BMI-SDS: age-specific BMI percentiles
