# Supplementary material for: Health related quality of life associated with extreme obesity in adolescents – results from the baseline evaluation of the YES-study
Source: Health Qual Life Outcomes. 2020 Mar 5;18:58. doi: 10.1186/s12955-020-01309-z (PMC7059717; doi:10.1186/s12955-020-01309-z)
Supplement: Supplementary file 5 — Additional file 5: Table S5. Linear regression analysis of the association of BMI-SDS with continuous measures of quality of life using two models but excluding participants from the job center in Essen. [file 12955_2020_1309_MOESM5_ESM.docx]

**Supplementary Table 5** Linear regression analysis of the association of BMI-SDS with continuous measures of quality of life using two models but excluding participants from the job center in Essen

|  | Variable | **EQ-VAS** | **DCGM-31** | **KINDLᴿ obesity** |
| --- | --- | --- | --- | --- |
|  |  | Estimate [95% CI] | Estimate [95% CI] | Estimate [95% CI] |
| **Model A** | **BMI-SDS** | -5.70 [-10.63; -0.76] | **-8.01 [-12.0; -4.05]** | -4.70 [-8.80; -0.60] |
|  | **Age** | -1.30 [-2.30; -0.30] | **-1.19 [-2.26; -0.12]** | -0.47 [-1.66; 0.71] |
|  | **Gender (female)** | 0.32 [-4.48; 5.13] | **-8.07 [-11.85; -4.29]** | -9.53 [-13.43; -5.63] |
| **Model B** | **BMI-SDS** | -4.94 [-10.92; 1.05] | **-8.52 [-13.27; -3.77]** | -3.60 [-8.80; 1.60] |
|  | **Age** | -1.60 [-3.36; 0.15] | -0.56 [-1.98; 0.86] | -0.14 [-1.80; 1.52] |
|  | **Gender (female)** | 1.74 [-3.97; 7.46] | **-8.97 [-13.34; -4.61]** | -9.23 [-14.10; -4.49] |
|  | **Pretreatment of obesity** |  |  |  |
|  | No pretreatment | Ref. | Ref. | Ref. |
|  | Inpatient | -0.09 [-6.94; 6.76] | -3.67 [-8.96; 1.62] | -2.17 [-7.86; 3.52] |
|  | Outpatient | 1.67 [-5.63; 8.96] | 0.89 [-4.87; 6.64] | 3.81 [-2.26; 9.88] |
|  | **Comorbidities (yes)^1^** | -1.21 [-7.40; 4.99] | -0.76 [-5.50; 3.98] | 1.71 [-3.46; 6.87] |
|  | **Physical activity (yes)^2^** | 5.12 [-0.95; 11.20] | 4.41 [-0.34; 9.17] | 4.28 [-0.86; 9.42] |
|  | **Parental education^3^** |  |  |  |
|  | low | Ref. | Ref. | Ref. |
|  | medium | 1.00 [-6.05; 8.05] | 1.89 [-3.78; 7.56] | 1.94 [-4.08; 7.96] |
|  | high | 8.62 [1.26; 15.98] | 3.50 [-2.35; 9.35] | 5.84 [-0.45; 12.12] |
|  | **Migration background^4^** | -3.87 [-9.92; 2.17] | -0.56 [-5.37; 4.26] | -2.98 [-8.16; 2.21] |
|  | **Screen time (>4h)** | -3.74 [-9.70; 2.22] | **-5.19 [-9.84; -0.54]** | -5.59 [-10.60; -0.58] |

^1^ hypertension, dyslipidemia and dysglycemia

^2^ based on answers to the question “Do you exercise regularly?”;

^3^ low education: no school graduation, high school with apprenticeship; medium education: middle school apprenticeship; high education: grammar school with/without university attendance;

^4^ at least one parent born abroad and/or foreign citizen status

^a^ Both models were additionally adjusted for institutes.

Note: EQ-VAS: visual analogue scale; DCGM-31: DISABKIDS chronic generic module without considering the medication item; BMI-SDS: age-specific BMI percentiles
