# Supplementary material for: Health related quality of life associated with extreme obesity in adolescents – results from the baseline evaluation of the YES-study
Source: Health Qual Life Outcomes. 2020 Mar 5;18:58. doi: 10.1186/s12955-020-01309-z (PMC7059717; doi:10.1186/s12955-020-01309-z)
Supplement: Supplementary file 6 — Additional file 6: Table S6. Linear regression analysis of the association of obesity grade with continuous measures of quality of life showing the results of model B and using imputed values for missing covariables and adjusting for participant education instead of parental education. [file 12955_2020_1309_MOESM6_ESM.docx]

**Supplementary Table 6** Linear regression analysis of the association of obesity grade with continuous measures of quality of life showing the results of model B and using imputed values for missing covariables and adjusting for participant education instead of parental education

|  | Variable | **EQ-VAS** | **DCGM-31** | **KINDLᴿ obesity** |
| --- | --- | --- | --- | --- |
|  |  | Estimate [95% CI] | Estimate [95% CI] | Estimate [95% CI] |
| **Model B** | **Obesity grade** |  |  |  |
|  | I | Ref. | Ref. | Ref. |
|  | II | -3.94 [-10.11; 2.23] | **-5.40 [-10.16; -0.63]** | -3.45 [-8.27; 1.38] |
|  | III | -2.52 [-8.93; 3.89] | **-7.53 [-12.62; -2.43]** | -5.24 [-10.29; -0.19] |
|  | **Age** | -1.19 [-2.60; 0.22] | -0.65 [-1.76; 0.46] | 0.04 [-1.16; 1.24] |
|  | **Gender (female)** | -0.29 [-5.20; 4.63] | **-9.12 [-12.91; -5.34]** | **-10.10 [-13.96; -6.23]** |
|  | **Pretreatment of obesity** |  |  |  |
|  | No pretreatment | Ref. | Ref. | Ref. |
|  | Inpatient | 0.69 [-5.12; 6.50] | -2.24 [-6.64; 2.15] | -2.37 [-6.89; 2.15] |
|  | Outpatient | 3.96 [-2.26; 10.18] | 2.68 [-2.21; 7.56] | 2.88 [-1.97; 7.73] |
|  | **Comorbidities (yes)^1^** | -3.15 [-8.58; 2.27] | 0.26 [-3.92; 4.44] | 2.67 [-1.57; 6.91] |
|  | **Physical activity (yes)^2^** | 4.78 [-0.35; 9.91] | 2.44 [-1.60; 6.48] | 1.46 [-2.62; 5.54] |
|  | **Participant education^3^** |  |  |  |
|  | low | Ref. | Ref. | Ref. |
|  | medium | -1.01 [-7.10; 5.09] | 4.24 [-0.63; 9.11] | **5.99 [1.13; 10.86]** |
|  | high | 0.21 [-7.20; 7.62] | 6.35 [0.29; 12.41] | **6.53 [0.66; 12.40]** |
|  | **Migration background^4^** | **-5.58[-10.76; -0.39]** | -1.80 [-5.90; 2.30] | -2.50 [-6.63; 1.63] |
|  | **Screen time (>4h)** | -4.10 [-9.14; 0.94] | **-5.45 [-9.39; -1.51]** | **-5.91 [-9.87; -1.95]** |

^1^ hypertension, dyslipidemia and dysglycemia

^2^ based on answers to the question “Do you exercise regularly?”;

^3^ low education: no school graduation, high school with apprenticeship; medium education: middle school apprenticeship; high education: grammar school with/without university attendance;

^4^ at least one parent born abroad and/or foreign citizen status

^a^ Both models were additionally adjusted for institutes.

Note: Values in bold are significant at p < 0.05; EQ-VAS: visual analogue scale; DCGM-31: DISABKIDS chronic generic module without considering the medication item; Obesity Grade definitions: I: BMI 30 to 34.9 kg/m^2^; II: BMI 35 to 39.9 kg/m^2^; III: BMI ≥ 40 kg/m^2^
