# Supplementary material for: Health related quality of life associated with extreme obesity in adolescents – results from the baseline evaluation of the YES-study
Source: Health Qual Life Outcomes. 2020 Mar 5;18:58. doi: 10.1186/s12955-020-01309-z (PMC7059717; doi:10.1186/s12955-020-01309-z)
Supplement: Supplementary file 7 — Additional file 7: Figure S1. Comparison of mean health related quality of life measured with EQ-VAS, KINDL and DSGM-31 according to BMI group and age below 17 years or above. [file 12955_2020_1309_MOESM7_ESM.docx]

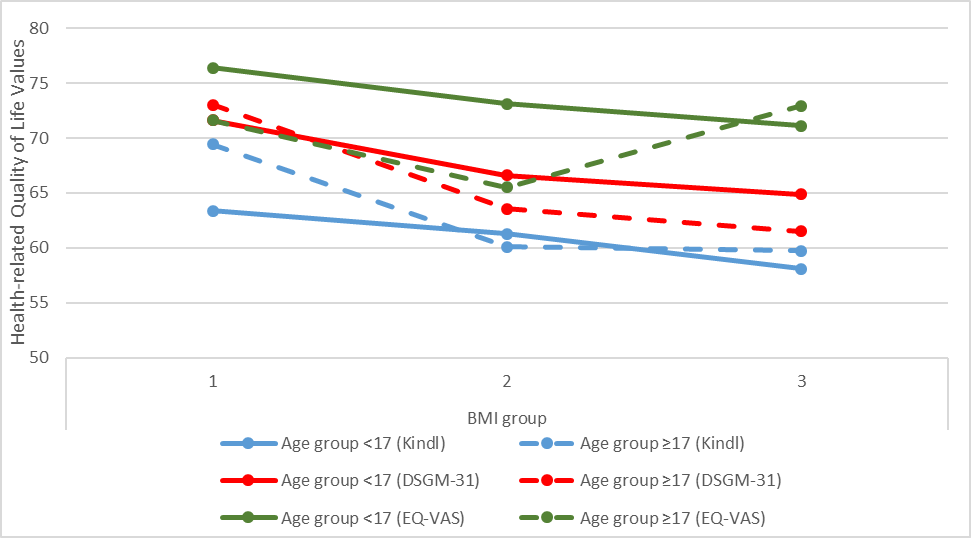


**Supplementary Figure 1:** Comparison of mean health related quality of life measured with EQ-VAS, KINDL and DSGM-31 according to BMI group and age below 17 years of age or above.

Note: Mean values adjusted for age, sex, pretreatment of obesity, comorbidity, physical activity, parental education, migration background, screen time. EQ-VAS: visual analogue scale; DCGM-31: DISABKIDS chronic generic module without considering the medication item; Obesity Grade definitions: I: BMI 30 to 34.9 kg/m^2^; II: BMI 35 to 39.9 kg/m^2^; III: BMI ≥ 40 kg/m^2^
